# Both projection and commissural pathways are disrupted in individuals with chronic stroke: investigating microstructural white matter correlates of motor recovery

**DOI:** 10.1186/1471-2202-13-107

**Published:** 2012-08-29

**Authors:** Michael R Borich, Cameron Mang, Lara A Boyd

**Affiliations:** 1 Department of Physical Therapy, Faculty of Medicine, University of British Columbia, 212-2177 Wesbrook Mall, Vancouver, British Columbia V6T 1Z3, Canada; 2 Brain Research Centre, University of British Columbia, Vancouver, British Columbia, Canada

**Keywords:** Diffusion tensor imaging, Stroke, Motor recovery, White matter, Integrity, Corpus callosum, Internal capsule

## Abstract

**Background:**

Complete recovery of motor function after stroke is rare with deficits persisting into the chronic phase of recovery. Diffusion tensor imaging (DTI) can evaluate relationships between white matter microstructure and motor function after stroke. The objective of this investigation was to characterize microstructural fiber integrity of motor and sensory regions of the corpus callosum (CC) and descending motor outputs of the posterior limb of the internal capsule (PLIC) in individuals with chronic stroke and evaluate the relationships between white matter integrity and motor function.

**Results:**

Standardized measures of upper extremity motor function were measured in thirteen individuals with chronic stroke. Manual dexterity was assessed in thirteen healthy age-matched control participants. DTI scans were completed for each participant. Fractional anisotropy (FA) of a cross-section of sensory and motor regions of the CC and the PLIC bilaterally were quantified. Multivariate analysis of variance evaluated differences between stroke and healthy groups. Correlational analyses were conducted for measures of motor function and FA. The stroke group exhibited reduced FA in the sensory (p = 0.001) region of the CC, contra- (p = 0.032) and ipsilesional (p = 0.001) PLIC, but not the motor region of the CC (p = 0.236). In the stroke group, significant correlations between contralesional PLIC FA and level of physical impairment (p = 0.005), grip strength (p = 0.006) and hand dexterity (p = 0.036) were observed.

**Conclusions:**

Microstructural status of the sensory region of the CC is reduced in chronic stroke. Future work is needed to explore relationships between callosal sensorimotor fiber integrity and interhemispheric interactions post-stroke. In addition, contralesional primary motor output tract integrity is uniquely and closely associated with multiple dimensions of motor recovery in the chronic phase of stroke suggesting it may be an important biomarker of overall motor recovery.

## Background

Deficits in motor function are common and a primary contributor to disability after stroke [1]. Approximately 80% of individuals with stroke experience hemiparesis and 55-75% experience varying degrees of chronic impairment in upper extremity motor function [1]. While altered activity in the sensorimotor cortices is a well-established component of motor recovery following stroke [2-5], recent evidence from studies using diffusion tensor imaging (DTI) demonstrate that changes in white matter may also be important [6-10]. The most commonly reported measure of white matter integrity from DTI is fractional anisotropy (FA). FA is a quantitative, unit-less measure of the directionality of water diffusion that indexes the microstructural properties of white matter [11]. Using this measure, relationships between white matter integrity and various measures of motor function have been demonstrated [6-10]. To date, most studies have focused on descending motor outputs of the posterior limb of the internal capsule (PLIC), while the motor and sensory regions of the corpus callosum (CC) have been studied less extensively. Moreover, a systematic evaluation of relationships between both ipsi- and contralesional PLIC integrity and a battery of assessments of motor recovery in the same patient cohort has yet to be undertaken. Thus, the present experiments were designed to explore differences in the integrity of motor and sensory fibers of the CC and motor output tracts of the PLIC between individuals with stroke and healthy age-matched controls, and to evaluate how these measures relate to multiple dimensions of motor recovery.

A primary role of the CC in the motor system is mediation of interhemispheric inhibition between the primary motor (M1) and sensory (S1) cortices to facilitate the performance of unimanual and coordinated bimanual movements [12,13]. Impairments in M1-M1 and S1-S1 interhemispheric inhibition appear to contribute to motor deficits after stroke [12,14], but the effects of stroke on the integrity of CC motor and sensory fibres and their relation to motor function have not been thoroughly described. Jang et al. [15] found that the presence of transcallosal fibers projecting from the unaffected corticospinal tract and descending towards the lesion were associated with poor motor function, but did not specifically evaluate the integrity of fibers within the callosum. Another study demonstrated that transcallosal M1-M1 fibre integrity was reduced in individuals with chronic stroke and that lower integrity of these tracts was associated with less improvement on the Wolf Motor Function Test (WMFT) following 5 consecutive days of non-invasive brain stimulation paired with physical therapy [16]. Interestingly, the integrity of these M1-M1 fibres was more strongly related to response to rehabilitation than integrity of the pyramidal tract and alternate descending motor tracts, but was not related to baseline level of motor function [16]. To our knowledge, no previous studies have specifically investigated alterations in CC sensory fibre integrity post-stroke and how such reductions may relate to motor recovery. In a longitudinal study, a geometric scheme employed by Witelson [17] was used to characterize temporal degeneration of the human CC over the period from 0–6 months post-stroke [18]. Gupta and colleagues [18] demonstrated region-specific reductions in CC integrity but did not evaluate how these reductions related to recovery. Further, the CC partitioning scheme used by Witelson [17] was predominantly based on experimental work in non-human primates [17] and more recent advances in DTI techniques allowed Hofer and Frahm [19] to re-evaluate the regional topographic partitioning of the CC in humans in vivo. The most striking finding of Hofer and Frahm [19] was that regions of the CC comprised of fibers projecting to the motor and sensory cortices were more posterior in the human CC than previously suggested by Witelson’s scheme [17]. This finding influenced development of a new modified scheme for partitioning the CC in humans [19]. This scheme more accurately characterizes the motor and sensory regions of the CC in humans, but has not been previously applied to evaluate CC integrity changes post-stroke or to examine how CC integrity relates to motor recovery post-stroke.

The integrity of corticofugal motor output projections has been more extensively studied in individuals with chronic stroke and has been found to relate to various measures of motor function [6-9]. Lower FA of the ipsilesional PLIC relative to the contralesional PLIC (i.e. FA asymmetry) relates to greater levels of physical impairment [8,9], lower levels of global motor function [9], and less strength in the affected hand [9]. Other studies have also reported that lower ipsilesional FA values are associated with greater impairments in global motor function [6] and hand dexterity [7]. These findings demonstrate that ipsilesional descending motor tract integrity is associated with level of post-stroke motor recovery. Schaechter and colleagues [7] also found that lower contralesional PLIC integrity was associated with reduced hand dexterity in individuals with chronic stroke, suggesting an important role of the contralesional descending motor outputs in mediating motor recovery post-stroke. Nevertheless, the relationship between contralesional PLIC integrity and other global measures of motor function and impairment post-stroke have not been previously reported. Additionally, the aforementioned data [6-9] were generated from separate investigations with different patient group characteristics, functional outcome measures, and research designs. A systematic evaluation of the relationships between both ipsi- and contralesional PLIC integrity and a battery of assessments of motor recovery, level of upper extremity impairment, strength and manual dexterity has yet to be undertaken in the same patient cohort.

The purpose of the present investigation was twofold. First, we evaluated differences in the integrity of callosal motor and sensory fibers using Hofer and Frahm’s modified geometric scheme [19], and the integrity of descending motor output tracts within the ipsi- and contralesional PLIC, between individuals with chronic stroke and healthy matched controls. Second, we examined relationships between microstructural integrity of these regions with multiple dimensions of motor function in individuals with chronic stroke. We hypothesized that FA would be lower in individuals with stroke than healthy age-matched controls in each of the regions evaluated. We also hypothesized that these measures of white matter integrity would be associated with all measures of motor recovery in individuals during the chronic phase of recovery from stroke.

## Results and discussion

### Comparisons between healthy and chronic stroke individuals

**Figure 1:**
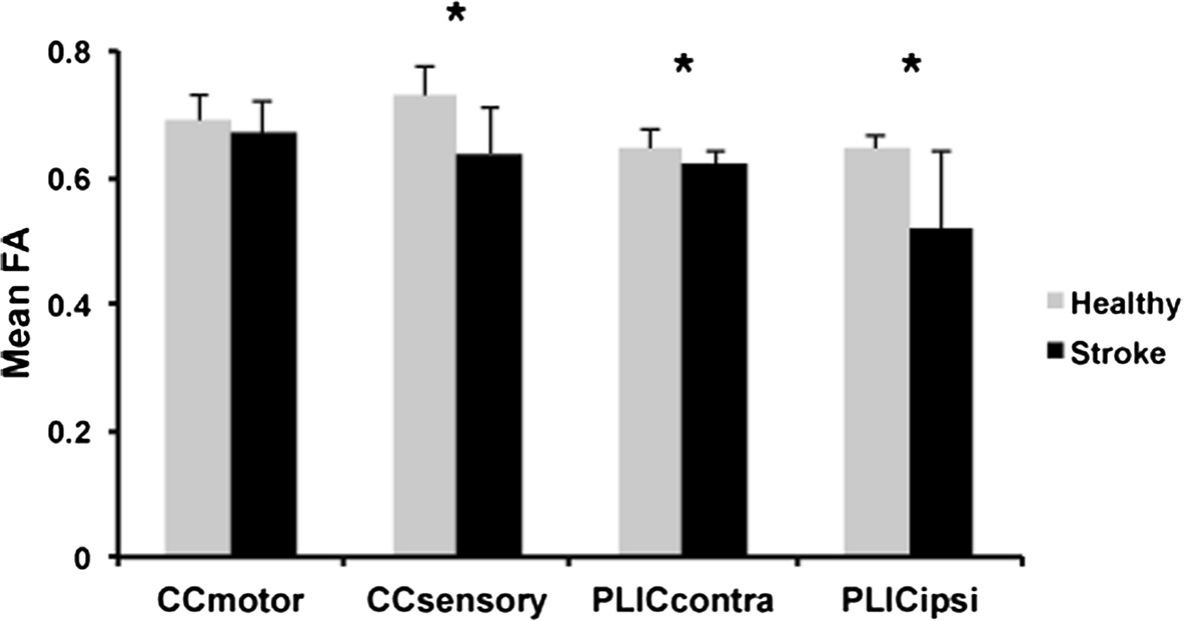
**White matter integrity in CC and PLIC in healthy individuals and patients with chronic stroke.** In patients with chronic stroke, reduced mean FA values were observed in both the contra- and ipsilesional PLIC and in the primary sensory region of CC. Error bars represent one standard deviation from the mean. *p < 0.05.

A significant main effect of group on white matter tract FA values was detected by the MANOVA (Wilks’ λ = 0.406, F_(4, 21)_ = 7.68, p = 0.001). Specifically, mean FA for the sensory region of the CC was significantly reduced compared to the healthy group (F_(1, 21)_ = 15.37, p = 0.001), but a significant difference was not observed between groups for CC motor region mean FA (F_(1, 21)_ = 1.48, p = 0.236) (Figure 1). For descending motor output tracts, mean FA was reduced in the contra- (F_(1, 21)_ = 5.16, p = 0.032) and ipsilesional PLIC (F_(1, 21)_ = 13.42, p = 0.001) in participants with stroke compared to healthy controls (Figure 1). Greater asymmetry in Box and Blocks test (BBT) performance was observed for individuals with stroke compared to the healthy group (t_(23)_ = −3.96, p = 0.001), indicating impaired manual dexterity in the affected limb in the chronic stroke group.

### Associations between assessments of motor function and white matter integrity

**Figure 2:**
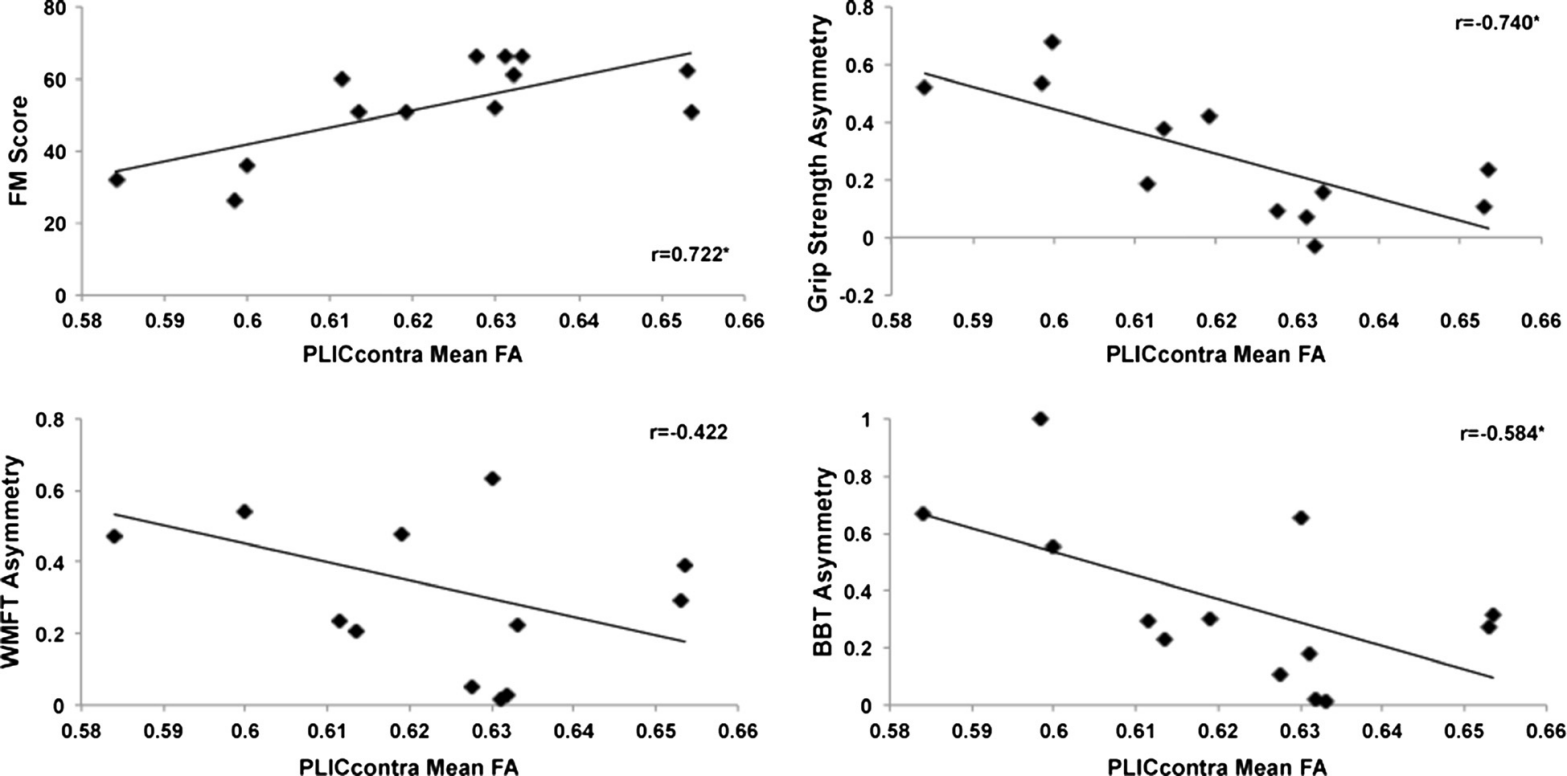
**Associations between contralateral PLIC integrity and multiple measures of motor function in patients with chronic stroke.** In patients with chronic stroke, significant correlations were observed between contralesional PLIC mean FA and FM score, grip strength asymmetry and BBT asymmetry. *p < 0.05.

In the healthy group, there were no significant correlations between age, white matter integrity, or motor performance (BBT score) (p > 0.05). In the stroke group, age was significantly correlated with Fugl-Meyer (FM) score (r = −0.571, p = 0.041), Wolf Motor Function Test (WMFT) asymmetry (r = 0.571, p = 0.042), grip strength asymmetry (r = 0.653, p = 0.021), BBT asymmetry (r = 0.687, p = 0.010), and contralesional PLIC FA (r = −0.668, p = 0.013). Post-stroke duration was correlated with FM (r = −0.723, p = 0.008), WMFT asymmetry (r = 0.643, p = 0.024), BBT asymmetry (r = 0.796, p = 0.002), and ipsilesional PLIC FA (r = −0.598, p = 0.040). For measures of white matter integrity, only FA of the contralesional PLIC was significantly correlated with measures of motor function (FM score: r = 0.722, p = 0.005; grip strength asymmetry: r = −0.740, p = 0.006; BBT asymmetry: r = −0.584, p = 0.036) (Figure 2).

### Summary of primary results

The integrity of CC sensory fibers, but not CC motor fibers, was reduced in individuals with chronic stroke compared to healthy controls. Additionally, contralesional PLIC integrity was associated with multiple measures of motor function post-stroke.

### Regional specificity of white matter integrity status in the corpus callosum is observed after stroke

We utilized the scheme put forth by Hofer and Frahm [19] to partition the human CC to evaluate differences in the integrity of regions of the CC comprised of interhemispheric motor and sensory tracts in individuals with chronic stroke and healthy individuals. This method of evaluating the CC [19] provides a more accurate measure of CC motor and sensory region integrity in humans post-stroke compared to previous work [18] utilizing a method predominantly based on primate research [17]. Moreover, by examining individuals with chronic stroke (>1 year post-stroke) we further extend previous research that only considered CC integrity changes during the acute phase of stroke recovery (0–6 months post-stroke).

Presently, individuals with chronic stroke demonstrated reduced integrity of the sensory CC region, but not the motor CC region, compared to healthy controls. Using Witelson’s scheme [17], Gupta and colleagues [18] also demonstrated region-specific reductions in CC integrity in the rostrum, genu, rostral body, anterior mid-body and splenium, but not the posterior mid-body and isthmus of the CC, in the acute phase of stroke recovery. The motor region of the CC in Hofer and Frahm’s modified scheme [19] corresponds to the posterior mid-body in Witelson’s scheme [17]. Thus, our finding of no difference in the CC motor region between individuals with chronic stroke and healthy controls is consistent with the previous finding of no change in posterior mid-body integrity throughout the acute phase of stroke [18]. On the other hand, this finding is in contrast to previous work indicating reduced integrity of trancallosal M1-M1 tracts in individuals with chronic stroke [16]. However, Lindenberg et al. [16] utilized tractography techniques to examine M1-M1 tracts, while the present study utilized a cross-sectional ROI approach to evaluate fibre integrity within the CC. In contrast to the CC motor region, the sensory region of the CC was significantly reduced in individuals with chronic stroke compared to healthy controls in the present study. The CC sensory region identified by Hofer’s scheme [19] corresponds to the anterior portion of the isthmus from Witelson’s scheme [17]. Gupta et al. [18] found no reduction in isthmus integrity during the acute phase of stroke. Thus, these contradictory findings may represent additional CC degeneration that occurs following the acute phase of stroke recovery. Alternatively, any reduction in integrity within this specific sensory area may have been undetected when measured as a small region within the larger isthmus.

Our finding of reduced sensory CC integrity in chronic stroke compliments previous work from our lab demonstrating that rehabilitation strategies designed to target S1-S1 interhemispheric interactions can have significant effects on motor system function in individuals with chronic stroke [20]. Repeated applications of inhibitory theta-burst stimulation over contralesional S1 prior to motor practice enhanced motor learning compared to sham stimulation in individuals with chronic stroke. Interestingly, individuals who received contralesional S1 stimulation demonstrated greater improvement in global motor function compared to those who received M1 stimulation [20]. Thus, these findings demonstrate that targeting S1-S1 interhemispheric interactions to facilitate ipsilesional S1 activity may offer unique strategies to facilitate post-stroke rehabilitation. The present finding of reduced CC sensory region integrity in individuals with chronic stroke provides additional evidence that the sensory system is affected in chronic stroke. Nevertheless, relationships between callosal sensory fibre integrity and motor function were not observed in the present study, suggesting that the integrity of this white matter region may not be particularly important for motor recovery. However, we feel that this is unlikely given the well-established role of inter-hemispheric interactions in mediating motor cortex excitability and function post-stroke [12,14,21-23]. Rather, we believe that the relatively small and homogenous sample of individuals presently studied may have limited the power for detecting such a relationship. Further, Lindenberg et al. [16] previously demonstrated that transcallosal M1-M1 tract integrity related to response to rehabilitation but not baseline motor function in individuals with chronic stroke. Thus, it is plausible that CC sensory fibre integrity may also be more strongly related to response to rehabilitation than to baseline measures of motor function. Future research with large heterogeneous samples examining callosal motor and sensory region integrity, neurophysiological measures of interhemispheric inhibition, bimanual motor tasks and response to rehabilitation will provide further insights into the functional relevance of post-stroke CC integrity changes.

### Contralesional descending motor outputs are associated with motor recovery after stroke

In addition to evaluating post-stroke callosal integrity changes, we also measured changes in the integrity of descending motor outputs within the PLIC. Previous work has demonstrated that reduced integrity of the contra- and ipsilesional PLICs are associated with a number of different functional measures in individuals with chronic stroke [6-10]; however, these findings have been compiled from multiple studies examining cohorts with different patient group characteristics, functional outcome measures, and research designs. Here, we evaluated the integrity of the contra- and ipsilesional PLIC and multiple measures of motor recovery in a single cohort of patients with chronic stroke. In our data, both contra-and ipsilesional PLIC integrity were decreased in individuals with chronic stroke compared to healthy age-matched controls. Degenerative changes of the contralesional PLIC were associated with greater level of physical impairment, lower hemiparetic upper extremity strength, and lower hand dexterity in chronic stroke. Similarly, Schaechter and colleagues [7] observed that reduced contralesional white matter integrity was associated with lower hand dexterity and finger tapping speed. The present study extends the findings of Schaechter et al. [7] by demonstrating that in addition to hand dexterity and finger tapping speed, contralesional white matter integrity is also associated with overall level of physical impairment and grip strength. Evidence from animal models of stroke recovery indicate that changes in white matter microstructure in the contralesional hemisphere including axonal sprouting, formation of new synapses and increased myelinating activity are potentiated by treatments that also improve motor recovery [24-26]. Thus, it is possible that similar processes operated in our cohort of individuals with chronic stroke, which may explain the relationships we note between contralesional PLIC integrity and motor function. These relationships were not observed in a matched group of healthy individuals suggesting a unique relationship between microstructural changes in descending motor output integrity and physical impairment, strength and manual dexterity in well-recovered individuals after stroke.

In contrast to previous work [7-9], ipsilesional motor output tract integrity was not significantly associated with measures of motor function in the present study. These differences may stem from differences in stroke participant characteristics in past studies. In the present study, the sample was fairly homogenous in terms of lesion location, level of recovery and, to a lesser extent, time since stroke onset. In other investigations, motor function and lesion location were more heterogeneous [7-9]. Additionally, the difference between the level of residual physical impairment in our patient sample and that of previous work may also contribute to this discrepancy. Our participants demonstrated higher FM scores indicating reduced upper extremity impairment and greater motor recovery in comparison to subjects in related investigations [8,27]. Taken together, these differences in patient characteristics may, in part, explain the observation of a relationship between contralesional, but not ipsilesional, tract integrity and motor function.

We also evaluated relationships between age and post-stroke duration of our stroke participants with measures of motor function and white matter integrity. Consistent with previous work [28], we observed that advancing age is associated with reduced motor function. Likewise, age was negatively correlated with contralesional PLIC integrity. We also observed that greater time since stroke was associated with lower motor function and lower ipsilesional PLIC integrity in individuals with chronic stroke. Previous work by Stinear et al. [8] indicated that longer time since stroke was associated with smaller improvements in FM score after motor practice. On the other hand, a recent systematic review determined that the evidence that time since stroke influences upper limb motor recovery is inconclusive [28]. The present findings suggest that motor function declines with greater time since stroke and that this decline may relate to degeneration of ipsilesional PLIC integrity. These data, in combination with previous work [8,28], demonstrate that simple demographic information may help explain the relationships observed between level of recovery and white matter degeneration.

### Limitations

Several limitations may impact the conclusions drawn from this study. Our sample of participants with stroke was relatively small and homogenous and thus may limit generalizability to the stroke population as a whole. We also limited our investigation of descending motor output tracts to a subsection of the motor output projection system previously shown to have reduced integrity following stroke [8,9] and shown to be reliable and sensitive in a subset of the present cohort of patients [29]. Other work has used alternative analysis techniques to evaluate this sub-section of tract but also other white matter regions within the brain [6,7,27,30]. Currently there is no gold standard for DTI analysis techniques in stroke and further work is needed to determine optimal imaging and analysis parameters.

## Conclusions

The present study demonstrates that the integrity of the sensory region of the CC is reduced in individuals with chronic stroke, but that the integrity of this region did not directly relate to the measures of motor function employed here. Future work is needed to elucidate relationships between CC integrity and interhemispheric sensorimotor interactions in the chronic phase of stroke. In addition, the present study demonstrates that contralesional descending motor output tract integrity, as well as demographic characteristics, are associated with multiple dimensions of motor function after stroke. Thus, contralesional motor output tract integrity may be an important biomarker of level of motor recovery in the chronic phase of stroke and may provide insights into future investigations of response to rehabilitation strategies.

## Methods

### Participants

Thirteen well-recovered individuals with chronic stroke (mean age ± SD: 63.8 ± 6.4) and thirteen age and gender-matched healthy control participants (mean age ± SD: 62.9 ± 7.4) were recruited from community and local postings. Participant characteristics are listed in Table 1. A subset of the individuals with stroke (n = 9) was part of a previous method development study [29]. Informed consent was obtained from each participant in accordance with the Declaration of Helsinki. University of British Columbia research ethics boards approved all aspects of the study protocol.

**Table 1:** Demographic information

**Stroke participants**																				
**Subject ID**	**Age (y)**	**Gender**	**Lesion location**	**Dominant Hand**	**PSD (mo)**	**MMSE**	**FM**	**WMFT Affected**	**WMFT Unaffected**	**WMFT Asym**	**Grip Affected**	**Grip Unaffected**	**Grip Asym**	**BBT Affected**	**BBT Unaffected**	**BBT Asym**	**CC FA**		**PLIC FA**	
																	**Motor**	**Sensory**	**Contra**	**Ipsi**
S01	65	M	R	R	20	28	51	3.24	1.15	0.48	16	39.5	0.42	30	56	0.30	0.75	0.71	0.62	0.56
S02	72	M	R	R	169	29	32	3.9	1.41	0.47	12	38	0.52	9	45	0.67	0.72	0.69	0.58	0.47
S03	59	F	R	R	42	30	61	1.09	1.03	0.03	19	18	-0.03	69	72	0.02	0.68	0.70	0.63	0.65
S04	72	M	R	R	101	27	52	5.28	1.19	0.63	NT	NT	NT	12	57	0.65	0.63	0.49	0.63	0.45
S05	74	M	R	R	65	29	36	4	1.2	0.54	8	42	0.68	12	42	0.56	0.66	0.67	0.60	0.59
S06	55	F	R	R	19	30	51	1.47	0.97	0.20	10	22	0.38	37	59	0.23	0.64	0.72	0.61	0.57
S07	59	M	L	R	38	28	62	1.71	0.94	0.29	37	46	0.11	34	59	0.27	0.67	0.60	0.65	0.57
S08	55	M	L	R	101	30	51	2.5	1.1	0.39	23	37	0.23	25	50	0.32	0.59	0.57	0.65	0.26
S09	64	M	R	R	29	30	66	0.66	0.64	0.02	26	30	0.07	49	70	0.18	0.75	0.73	0.63	0.52
S10	65	F	R	R	90	29	60	4.16	0.72	0.23	18	26	0.18	32	59	0.30	0.62	0.57	0.61	0.35
S11	68	F	R	R	136	30	26	4.5	0.81	0.84	6	20	0.54	0	63	1.00	0.68	0.62	0.60	0.47
S12	58	M	R	R	17	30	66	1.28	0.81	0.22	27.33	37.33	0.15	45	46	0.01	0.70	0.65	0.63	0.67
S13	63	M	R	R	28	29	66	1	0.9	0.05	25	30	0.09	51	63	0.11	0.62	0.57	0.63	0.54
Mean ± SD: 63.76 ± 6.42					68.08 ± 50.99	29.15 ± 0.99	52.31 ± 13.44	2.45 ± 1.56	2.45 ± 1.56	0.99 ± 0.22	18.94 ± 9.17	32.15 ± 9.17	0.28 ± 0.22	31.15 ± 19.61	57 ± 19.61	0.35 ± 0.29	0.67 ± 0.05	0.64 ± 0.07	0.62 ± 0.02	0.52 ± 0.12
**Healthy participants**																				
**Subject ID**	**Age (y)**	**Gender**	**Dominant Hand**	**BBT Non- dominant**	**BBT Dominant**	**BBT Asym**	**CC FA**		**PLIC FA**											
							**Motor**	**Sensor**	**Contra**	**Ipsi**										
S101	64	F	R	55	63	0.07	0.67	0.75	0.58	0.60										
S102	72	F	R	68	58	−0.08	0.70	0.75	0.64	0.63										
S103	67	F	R	68	77	0.06	0.63	0.66	0.68	0.66										
S104	63	M	R	77	74	−0.02	0.67	0.72	0.61	0.60										
S105	60	F	R	56	61	0.04	0.67	0.75	0.67	0.67										
S106	51	M	R	82	75	−0.04	0.72	0.71	0.64	0.64										
S107	68	M	R	67	63	−0.03	0.68	0.76	0.65	0.63										
S108	69	M	R	68	72	0.03	0.62	0.64	0.62	0.64										
S109	48	F	R	77	77	0.00	0.70	0.73	0.63	0.65										
S110	67	F	R	52	54	0.02	0.73	0.76	0.63	0.64										
S111	55	F	R	NT	NT	NT	0.75	0.80	0.68	0.68										
S112	68	F	R	53	65	0.10	0.72	0.73	0.68	0.67										
S113	66	M	R	47	55	0.08	0.72	0.74	0.69	0.67										
Mean ± SD:62.92 ± 7.36				64.17 ± 11.35	66.17 ± 8.50	0.02 ± 0.05	0.69 ± 0.04	0.73 ± 0.04	0.65 ± 0.03	0.64 ± 0.02										

PSD: Post-stroke duration, MMSE: Mini-Mental Status Exam, BBT: Box and Blocks test, FM: Fugl-Meyer, WMFT: Wolf motor function test, Asym: Asymmetry, Contra: Contralesional, Ispi: Ipsilesional, M: Male, F: Female, L: Left, R: Right, SD: Standard deviation, y: years, mo: months, NT: not tested.

### Research design

Each participant completed motor function assessments and magnetic resonance imaging (MRI) on separate days.

### Functional assessments

Participants in the stroke group completed a battery of assessments to comprehensively measure upper extremity motor impairment, motor function, grip strength and manual dexterity. These assessments were administered by a licensed physical therapist. Physical impairment level of the involved arm was assessed using the upper extremity motor portion of the FM assessment (range of scores 0–66) containing 33 items scored from 0–2 with higher scores indicating less physical impairment [31]. The WMFT has been shown to be a reliable and valid comprehensive assessment of upper extremity motor function [32]. Testing consisted of fifteen timed movement tasks and two tests of strength. Movement time for each task was averaged over three trials and median movement time was calculated across all tasks. Maximum grip strength was averaged over three trials using a calibrated handheld dynamometer. The BBT reliably measures hand dexterity in stroke [33]. For the BBT, participants grasped a 2.54 cm^3^ wooden block on one side of a divided box using the thumb and index finger and released it on the other side. Performance was quantified by number of blocks transferred in 60s [34]. Individuals in the healthy group completed the BBT to provide a comparison of motor performance between groups. The WMFT, grip strength assessment and BBT were conducted bilaterally for each participant in the stroke group. Asymmetry scores were calculated for each participant:

(1) $$\frac{{\text{WMFT}}_{\text{aff}}-{\text{WMFT}}_{\text{unaff}}}{{\text{WMFT}}_{\text{aff}}+{\text{WMFT}}_{\text{unaff}}}\frac{{\text{Grip}}_{\text{unaff}}-{\text{Grip}}_{\text{aff}}}{{\text{Grip}}_{\text{unaff}}+{\text{Grip}}_{\text{aff}}}\frac{{\text{BBT}}_{\text{unaff}}-{\text{BBT}}_{\text{aff}}}{{\text{BBT}}_{\text{unaff}}+{\text{BBT}}_{\text{aff}}}$$

Asymmetry values could range from −1.0 to +1.0 with positive values indicating greater impairment and negative values indicating less impairment of the affected upper extremity compared to the less affected extremity. Values of 0.0 were indicative of symmetrical performance between extremities. Functional assessment scores for each participant are listed in Table 1.

### MR data acquisition

MR acquisition was conducted at the UBC MRI Research Centre on a Philips Achieva 3.0 T whole body MRI scanner (Phillips Healthcare, Andover, MD) using an eight-channel sensitivity encoding head coil (SENSE factor = 2.4) and parallel imaging. A high-resolution anatomical scan (TR = 12.4 ms, TE = 5.4 ms, flip angle θ = 8°, FOV = 256 mm, 170 slices, 1 mm thickness) was collected. A diffusion weighted scan was conducted with a single shot echo-planar imaging (EPI) sequence (TR = 7465 ms, TE = 75 ms, FOV = 212 × 212 mm, 60 slices, 2.2 mm slice thickness, voxel dimension =2.2^3^ mm). Diffusion weighting was applied across 15 independent non-collinear orientations (b = 1000 s/mm^2^) along with a non-weighted diffusion weighted image acquired (b = 0). A gradient table was used for subsequent data analysis, computed using parameters of the diffusion-weighted images [35].

### MR data processing

Prior to tensor calculation, the quality of the raw images were visually inspected for excessive motion artifact or instrumental noise using a slice-by-slice procedure; if an image was deemed corrupt, it was removed prior to final tensor calculation [36]. Less than 1% of images were removed across all subjects. After tensor calculation, FA maps were produced based on the magnitude of diffusivity in three defined orientations and the mean diffusivity of each within a given tensor [37]. Color-coded orientation maps were used to visualize the principal fiber orientation within each pixel (red: right-left, blue: superior-inferior, green: anterior-posterior).

**Figure 3:**
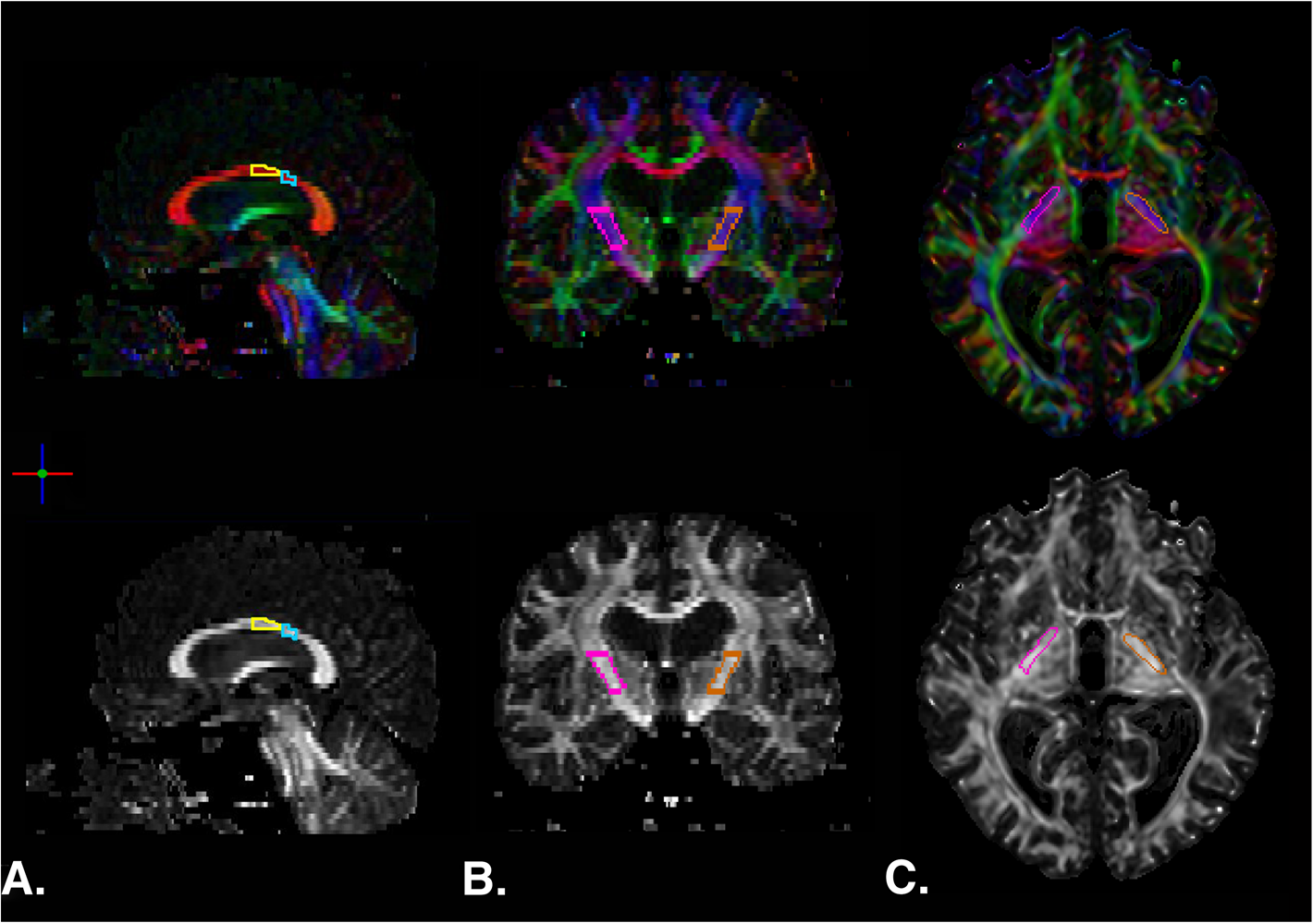
**DTI-derived color and FA maps depicting region of interest drawings from a representative subject in the stroke group. A.** Sagittal view of CC motor (yellow) and sensory (blue) regions as identified using Hofer and Frahm’s modified scheme [18]. **B.** Coronal view of right (pink) and left (orange) PLIC ROIs. **C.** Axial view of PLIC ROIs at level of the anterior commissure.

The ROIEditor software program ( http://www.MriStudio.org) was used to perform manual quantification of the integrity of the segments of the CC comprised of motor and sensory fibers [19] and the PLIC [8]. The cross-sectional regions of interest (ROIs) were delineated consulting the FA and color maps produced and standard white matter atlas [38]. ROIs for the CC were delineated in the mid-sagittal plane and in the adjacent five slices to the right and to the left using the scheme proposed by Hofer and Frahm [19]. A geometric baseline for the CC was defined by connecting the most anterior and posterior points of the CC. ROIs were then delineated over the regions comprising fibers projecting to motor and sensory cortices. The motor region was defined as the posterior half minus the posterior third of the callosum and the sensory region as the posterior one-third minus the posterior one fourth of the callosum [19] (Figure 3A). No lesions penetrated the callosum. The PLIC was delineated bilaterally, beginning at the level of the anterior commissure and terminating at the inferior border of the corona radiata [8] (Figure 3B). Lesions penetrating the PLIC were not excluded from the drawing procedure. Complete disruption of the affected PLIC was not observed for any participant. Mean FA values for each region (CC motor, CC sensory, ipsilesional PLIC, and contralesional PLIC) were then computed removing pixels with zero or negative FA values and the mean FA from the remaining voxels within the manually defined ROI mask were used for statistical analyses.

### Statistical analyses

A between-groups multivariate analysis of variance (MANOVA) assessed differences in white matter integrity between healthy individuals and individuals with stroke. The dependent variables were FA obtained from the various ROIs drawn: CC motor region, CC sensory region, ipsi- and contralesional PLIC. For participants in the healthy group, FA of “ipsi- and contralesional” PLIC were quantified from the non-dominant and dominant hemisphere, respectively. Additionally, an independent samples t-test was conducted to evaluate differences between groups in BBT asymmetry.

After assessing between-group differences in motor function and white matter integrity, the groups were evaluated individually to study the relationships between white matter integrity and baseline measures of motor function. Simple bivariate parametric correlation analyses between measures of age and post-stroke duration, measures of motor function, and white matter integrity were conducted. For each statistical test, significance level was: uncorrected p < 0.05. All statistical procedures were conducted using SPSS software (SPSS 19.0).

## Authors’ contributions

LAB conceived and participated in the design of the study. MRB performed data processing, statistical analysis and drafted the manuscript. CM participated in data processing and analysis and helped draft the manuscript. All authors contributed to interpretation of results. All authors read and approved the final manuscript.
